# Increasing Nitrogen Losses Due to Changing Food Consumption Patterns in Bayannur City, China

**DOI:** 10.3390/foods12040752

**Published:** 2023-02-08

**Authors:** Yihang Liang, Yanqin Zhang, Yuyue Wang, Xinggong Kong, Zucong Cai, Yanhua Wang

**Affiliations:** 1 School of Geography, Nanjing Normal University, Nanjing 210023, China; 2 Jiangsu Center for Collaborative Innovation in Geographical Information Resource Development and Application, Nanjing 210023, China; 3 Key Laboratory of Virtual Geographic Environment, Ministry of Education, Nanjing Normal University, Nanjing 210023, China

**Keywords:** food consumption patterns, food supply, nitrogen losses, environmental influence, Bayannur City

## Abstract

Increasing urbanization and affluence have led to changes in food consumption patterns. The application of nitrogen (N) fertilizers ensures food security but also leads to environmental pollution due to N losses, through processes such as acidification, eutrophication, and greenhouse gas emissions. To clarify whether changes in food consumption patterns could increase N losses and to explore sustainable food system pathways, this study integrated the Chinese Food System Dashboard and the Nutrient Flows in Food Chains, Environment and Resources Use model to quantify and compare the link between food consumption and N losses in different agricultural regions using a case study of Bayannur City in the Yellow River Basin from 2000 to 2016. During the study period, Bayannur’s food consumption pattern changed from a “high carbohydrate and pork pattern” to a “high fiber and herbivore pattern”, which represents a shift from low to high N consumption. The per-capita food consumption decreased by 11.55% from 425.41 kg cap^−1^, whereas the per-capita N losses increased by 12.42% from 35.60 kg N cap^−1^. The average share of the plant-oriented and animal-oriented food supply in these losses was 53.39% and 46.61%, respectively. There were differences in the food consumption patterns and N losses in Bayannur’s farming, farming–pastoral, and pastoral regions. The changes in N losses were most significant in the pastoral region. The N losses to the environment increased sharply by 112.33% from 22.75 g N cap^−1^ over the past 16 years. The low level of economic development in Bayannur resulted in a shift in the food consumption pattern to a high N consumption. Four measures to protect food security and reduce the food N cost were proposed: (1) increasing the wheat planting area and maintaining the existing corn one; (2) expanding the scale of high-quality alfalfa planting; (3) enhancing the area of oat grass and wheat replanting; and (4) using modern planting technology.

## 1. Introduction

Zero hunger and responsible consumption and production are 2 of the 17 Sustainable Development Goals of the United Nations [1]. With the continued pace of urbanization in developing countries, people have become increasingly disconnected from food production [2,3,4,5]. Nutritional recommendations or dietary guidelines released by governments have also encouraged consumers to pay more attention to food nutrition and health [6,7]. To ensure adequate food consumption for humans and livestock, nitrogen (N) fertilizers are widely used on farms to increase crop production and quality [8,9,10]. However, only a small share of the N used in crop and animal production reaches the plates of consumers [8,11,12]. Large amounts of N are released into the environment, which compromises air, water, and soil quality, resulting in a deterioration in ecosystem services and human health [13,14,15,16].

Crop production and livestock production increased by 47.75% and 49.49%, respectively during 2000−2021 in China [17], which lifted millions of people out of poverty and hunger and also reshaped food consumption patterns [18,19]. However, the subsequent changes in N load in various media associated with their environmental effects can not be underestimated [20,21,22,23,24,25]. The 2020 China Ecological Environment Status Bulletin revealed that N pollution derived from agricultural sources accounts for 50% of all environmental pollution. In addition, the Food and Agriculture Organization of the United Nations (FAO) also reported in the COP 26 UN Climate Change Conference that from 1990 to 2019, the global agricultural grain system’s greenhouse gas emissions increased by 17%, with China’s total emissions ranking among the top five. Because more and more governments have a significant focus on food security and responsible production [26,27], linking food consumption patterns with environmental impacts could be beneficial to the development of human society.

The ecological conservation and high-quality development of the Yellow River Basin (YRB) is a national strategy that was proposed in 2019. Unfortunately, recent global and regional forecasts indicate that the upper YRB is on a warm-dry trend in the 21st century [28]. The increasingly unfavorable planting conditions have led to the dual challenge of food safety and ecological conservation [29,30]. The current low N use efficiencies in the YRB have resulted in the local N losses being higher than in other regions. According to the results of the second national census on pollution in China, the total N (TN) produced by non-point sources in Inner Mongolia accounted for 70.19% of the total in the upper YRB, which accounted for 20.22% of the YRB as a whole. In summary, an investigation of whether changes in food consumption patterns can increase N losses will be meaningful for future N management and the adjustment of food consumption patterns.

In 2019, the policy that “the Hetao Irrigation District in Inner Mongolia should develop modern agriculture, raise the quality of agricultural products and contribute to national food security” was proposed. Bayannur City is located in the golden plantation belt of the Hetao Irrigation District of the upper YRB. The 598,995 ha of cultivated soil in the region is irrigated silt soil from the Yellow River, which permits the cultivation of a variety of crops [31,32]. Wheat, corn, and sunflower are the dominant crops, with sunflower production being the main income-generating activity for farmers. The continental monsoon climate has also enabled Bayannur to develop a livestock industry, and the city is responsible for the largest livestock exports in Inner Mongolia. Bayannur, therefore, can serve as an example of improved N management in Chinese cities with high N losses.

Improvements in the quality of life in human societies will inevitably lead to changes in food consumption patterns [33,34]. In addition, consumers’ dietary habits and whether they live in farming or pastoral regions, can also influence food consumption patterns [35]. This is because different land use and cropping structures increase regional disparities and have different environmental impacts. Previous studies have used environmental footprint approaches [36,37,38,39] and life cycle assessments [40,41,42] to assess the various impacts of agriculture. Studies have mostly been conducted at national, provincial, and watershed scales, and have provided meaningful insights from a production perspective [43,44]. Previous studies have shown that excess carbon (C), N, and phosphorus (P) losses have resulted in many environmental problems, such as acidification, eutrophication, and greenhouse gas emissions [45,46]. Overall, there are no models suitable for application at municipal scales and there has been no comprehensive assessment of the environmental impact of food consumption in different agricultural regions from a consumer perspective.

This study aimed to: (1) construct the model to link food consumption and N losses; (2) portray the food consumption patterns across the study region; (3) estimate N losses associated with changes in food consumption patterns; and (4) clarify optimal food and N management to minimize the environmental N losses.

## 2. Materials and Methods

### 2.1. Description of the Study Area

**Figure 1:**
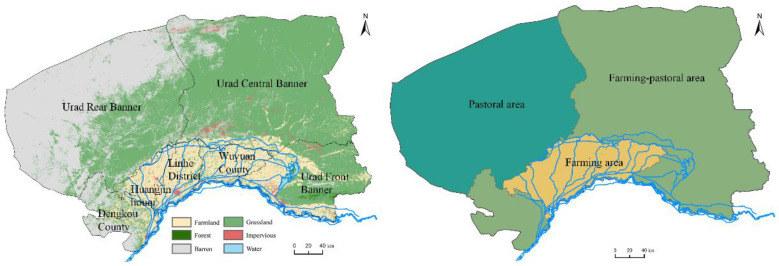
Location and land use cover change maps of the three agriculture regions in Bayannur. Data from China Mapping and Geographic Information Bureau (http://www.sbsm.gov.cn/, accessed on 1 November 2022).

Bayannur City is an extremely important city in Inner Mongolia, China, that contains the Urater Grassland, Yin Mountains, and Hetao Irrigation District (Figure 1). There are seven counties in Bayannur, which had a registered population of 1,538,715 people in 2020. Based on land use and agricultural differences, the government has divided them into three regions: farming, farming–pastoral, and pastoral regions. The farming region includes three counties (Wuyuan County, Linhe District, and Hanging Rear Banner), which are located in the Hetao Irrigation District and have a combined population of 1,024,382 people. The farming–pastoral region includes Dengkou County, Urad Front Banner, and Urad Middle Banner, which have a combined population of 460,181 people. Urad Back Banner in the pastoral region has a population of only 53,946 people. Bayannur is a low and middle-income city with a GDP per capita (CNY 56,600 per person) lower than the national level (CNY 71,800 per person) in 2020.

### 2.2. The Food Supply and N Loss Calculation

As a basic framework for linking food consumption and food supply, the Chinese government established the Chinese Food System Dashboard (CFSD) to actively address the issues of the inability to link food consumption and supply. The CFSD advocates agroecology and explores sustainable food supply pathways by considering synergies in a consistent manner [18]. The Nutrient Flows in Food Chains, Environment and Resources Use (NUFER) model can quantify N losses by adjusting parameters and N flow processes [11,47]. Therefore, combining the two models allows the link between food consumption and N losses in different agricultural regions to be quantified and compared.

**Figure 2:**
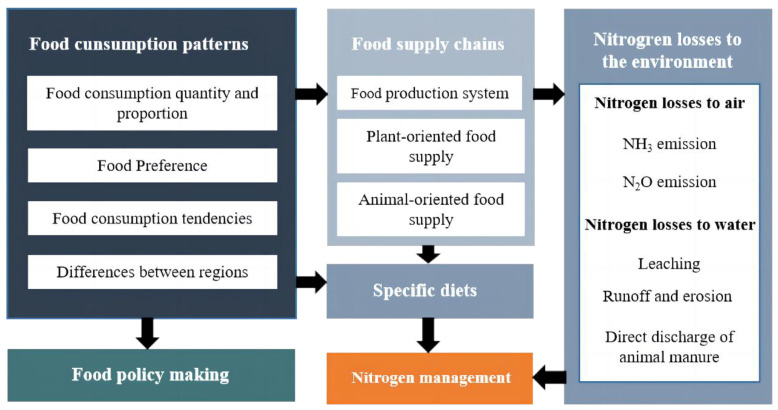
The CFSD and NUFER framework for analyzing the food consumption patterns and N losses.

To quantify the relative contribution of different foods to the N losses, the NUFER model needs to be integrated into the CFSD (Figure 2). The models were integrated using an embedded method, after which the integrated model was divided into three parts. First, we calculated the food consumption in Bayannur. We measured food consumption based on statistical yearbooks and Bayannur government reports. The food was divided into plant-oriented food (cereals, beans, vegetables, oils, and fruits) and animal-oriented food (pork, mutton, poultry, and fish). Beef consumption was too low to be included in the animal-oriented food. Second, we used the updated CFSD to estimate the food supply, which was back-projected using food consumption, involving parameters such as the proportion of grains that were consumed, and the proportion of meat in animals. Taking care to distinguish between plant-oriented and animal-oriented food in the food supply chain. Third, the integrated model was used to estimate N losses. In the present study, the N losses in food production were largely derived from crop straw, animal excreta, and feeding losses. The parameters in the model, such as the cultivation and livestock breeding coefficients, were updated to fit Bayannur.

Notably, even though the Hetao Irrigation District was rich in products, economic development and trade remained limited due to its inland location. In 2016, the GDP per capita in Bayannur was only 75.63% of the average in Inner Mongolia. Meanwhile, Bayannur was committed to building the China–Mongolia–Russia Economic Corridor, and a large amount of high-quality coal and copper concentrates were imported from the northern trade ports during the study period, while fewer agricultural products were imported. Large-scale sheep breeding has resulted in abundant wool production, which has made wool the main exported agricultural product. Therefore, the integrated model was based on the self-production of food in the study area, where the effects of food import and export were negligible. Especially, only local production was considered in the calculation of food supply, but it should be traced separately in the farming, farming–pastoral, and pastoral regions.

The specific and detailed equations used in the calculations were as follows:

(1) Estimation of food supply

Food supply refers to the production of crops and livestock to meet people’s food consumption requirements. We assumed that the reactive food supply from food production, such as the storage, transportation, processing, packaging, and retail sectors could be ignored. The food supply was calculated as follows:

(1) $$Food_{S}=Food_{S-pla}+Food_{S-ani}$$

(2) $$Food_{S-pla}=\overset{m}{\underset{i=1}{\sum }}\left(Food_{C-pla}/P_{rations-food}/P_{grains-rations}\right)$$

(3) $$Food_{S-ani}=\overset{n}{\underset{j=1}{\sum }}\left(Food_{C-ani}/P_{M}\right)$$

where $Food_{S}$, $Food_{S-pla}$, and $Food_{S-ani}$ represent the total food supply (kg), plant-oriented food supply (kg), and animal-oriented food supply (kg), respectively; i and j refer to different types, respectively; $Food_{C-pla}$ and $Food_{C-ani}$ are plant- and animal-oriented consumption (kg), respectively; $P_{rations-food}$ is the proportion of rations used as food (%); $P_{grains-rations}$ is the proportion of grains used as rations (%); and $P_{M}$ is the proportion of meat in animals (%).

(2) Estimation of N loss

The N losses to the environment from the food supply were calculated using the modified NUFER model [11]. The improvements in the model made it more applicable to Bayannur and also made it easier to calculate the N losses due to plant- and animal-oriented food. Equations (4)–(8) were applied.

(4) $$N_{Loss}=N_{L-pla-air}+N_{L-pla-water}+N_{L-ani-air}+N_{L-ani-water}$$

(5) $$N_{L-pla-air}=\overset{m}{\underset{i=1}{\sum }}\left[\left(Food_{S-pla}/U_{pro}\right)\times N_{application}\times \left(P_{NH_{3}}+P_{NO_{X}}\right)\right]$$

(6) $$N_{L-pla-water}=\overset{m}{\underset{i=1}{\sum }}\left[\left(Food_{S-pla}/U_{pro}\right)\times N_{application}\times \left(P_{runoff}+P_{leaching}\right)\right]$$

(7) $$N_{L-ani-air}=\overset{n}{\underset{j=1}{\sum }}\left(Food_{S-ani}\times N_{manner}\times P_{manner-volatilization}\right)$$

(8) $$N_{L-ani-water}=\overset{n}{\underset{j=1}{\sum }}\left(Food_{S-ani}\times N_{manner}\times P_{manner-water}\right)$$

where $N_{Loss}$ represents the N losses quantity (kg); $N_{L-pla-air}$ and $N_{L-pla-water}$ refer to the N losses into air and water during the production of plant-oriented food, respectively (kg); $N_{L-ani-air}$ and $N_{L-ani-water}$ represent the N losses into air and water during the production of animal-oriented food, respectively (kg); $U_{pro}$ is the production of the crop (kg ha^−1^); $N_{application}$ is the N input to crop from all N resources (kg); $P_{NH_{3}}$ and $P_{NO_{X}}$ represent the proportion of N losses to air via ammonia (NH_3_) and N oxides (NO_X_), respectively (%); $P_{runoff}$ and $P_{leaching}$ represent the proportion of N losses to water via surface and leaching, respectively (%); $N_{manner}$ is the N content of manure (%); $P_{manner-volatilization}$ is the proportion of manure volatilization (%); $P_{manner-water}$ represents the proportion of manure entering the water (%); and the other parameters are the same as those in Equations (1)–(3).

### 2.3. The Food N Cost Calculation

The food N cost (FNC) refers to the amount of new N input (kg) to the model when 1 kg of food N is consumed by the consumer in the region and reflects the resource and environmental cost of food consumption in the region. The FNC was calculated as follows:

(9) $$FNC=\frac{N_{input}}{N_{pla}+N_{ani}}$$

where $N_{pla}$ and $N_{ani}$ represent the total N in plant- and animal-oriented food, respectively (kg); and $N_{input}$ is the N input from all N resources (kg).

### 2.4. Data Sources and Uncertainty Analysis

In this study, the data were divided into three categories. First, statistical data for population, food consumption, cultivated area, crop yields, and land use were obtained from the *Bayannur Statistical Yearbook* (2000−2016), FAO (https://www.fao.org/home/en, accessed on 1 September 2022), World Health Organization (https://www.who.int/, accessed on 1 September 2022), and Our World in Data (https://ourworldindata.org/, accessed on 1 September 2022). Second, the coefficients of the proportion of rations used as food, the proportion of grains used as rations, the proportion of meat in animals, the proportion of N losses to air via NH_3_ and NO_X_, the proportion of N losses to water via surface leaching, the proportion of manure volatilization, the proportion of manure entering the water, and other basic parameters were sourced from the literature [11,12].

To determine the uncertainty in N losses due to the above coefficients, a sensitivity analysis was performed with an error propagation equation used in statistical analyses [11]. As shown in Equation (10), the change rate of the parameters was taken as ±10%.

(10) $$I=\frac{\Delta Y_{i}}{Y_{i}}/\frac{\Delta X_{i}}{X_{i}}$$

where I indicates the sensitivity index; $X_{i}$ is a parameter of i;$\;Y_{i}$ is the output of the model; and $\Delta X_{i}$ and $\Delta Y_{i}$ represent the variation of the parameters and outputs, respectively. The larger the absolute value of the index, the more flexible the sensitivity of the parameter to the model, and the more prominent its role in the model.

The sensitivity analysis results revealed that no parameter was extremely sensitive to the N losses. The three parameters of cereals N application, vegetables N application, and weight of the pig, significantly influenced the model performance. We set up different uncertainty ranges for these parameters (Table 1). An uncertainty analysis was conducted with an error propagation equation used in statistical analyses. The results showed that all parameters had small coefficients of variation. The weight of the pig was obtained from social survey data, and the other parameters were obtained from the published literature, which assured the accuracy of the parameters.

## 3. Results and Discussion

### 3.1. Food Consumption Patterns

**Figure 3:**
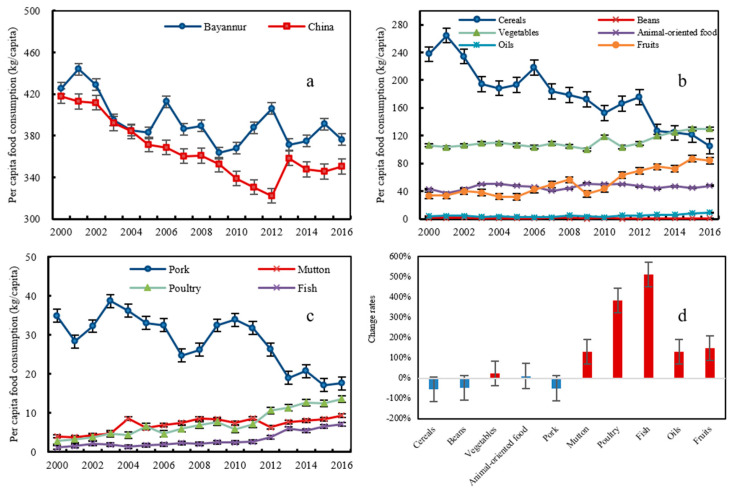
Changes in the per capita food consumption in Bayannur from 2000–2016: (**a**) per-capita food consumption in Bayannur and China; (**b**) per-capita consumption of all individual food items in Bayannur; (**c**) per-capita animal-oriented food consumption in Bayannur; and (**d**) changes in the consumption of all food items.

From 2000 to 2016, the per-capita food consumption in Bayannur displayed a decreasing trend. It decreased by 14.60% from 425.41 kg cap^−1^ in 2000 to 363.29 kg cap^−1^ in 2009, and then fluctuated within the range of 363.29–405.88 kg cap^−1^ (Figure 3). The overall trend was similar to the trend in per-capita food consumption in China. In 2009, Bayannur experienced a series of serious meteorological disasters. A severe drought resulted in the death of 438,003 head of large livestock in the pastoral regions and affected a total area of 2,044,578 ha; insect and rat diseases affected a total area of 120,000 ha in the farming–pastoral region; and dust storms and hailstorms affected more than 45,000 ha of crops in the farming region, with 9644 ha of crops lost. The lack of food production at this time explained why food consumption fell to its lowest level in 2009.

During the study period, the per-capita cereal consumption decreased from 237.40 to 104.64 kg cap^−1^, while vegetable and fruit consumption increased from 106.04 and 33.66 kg cap^−1^ by 22.60% and 149.35%, respectively. Although the total per-capita livestock product consumption changed little, pork consumption decreased by 49.60%, while poultry and mutton consumption increased by 132.01% and 383.63%, respectively. Per-capita sunflower oil consumption increased from 4.04 to 9.27 kg cap^−1^. Unlike the other foods, per-capita bean consumption was small and fluctuated within the range of 0.41–1.66 kg cap^−1^.

In 2000, one of the dominant characteristics of food consumption was that cereals and pork were the major food items consumed, accounting for 64.00% of total consumption. This consumption pattern was described as the “high carbohydrate and pork pattern” and was highly dependent on the grains produced by the farming system. However, it was found that the proportional consumption of cereals and pork decreased from 64.00% in 2000 to 32.48% in 2016, suggesting that these food items had become “unpopular”. People were no longer satisfied with cereals and pork and shifted to coarse fiber, poultry, and mutton, which together increased from 34.45% to 62.95% of total consumption. This consumption pattern reflected an improving economy and was described as the “high fiber and herbivore pattern”. It was reliant on farmland for cash crops and grassland that produce high N losses food items [11].

Similar changes in food consumption patterns also occurred at the national scale during the study period. Between 2000 and 2016, cereal consumption as a proportion of all food items decreased from 59.88% to 44.88%, while the proportional consumption of vegetables, fruits, and animal-oriented food increased from 25.54%, 4.38%, and 6.71% to 26.12%, 10.51%, and 15.19%, respectively. Changes in the human population and gross domestic production (GDP) were considered to be the main driving forces of these changes, although research and technology development and the behavior of food chain actors (suppliers, processing industry, and retail) also played an important role. The representative global food consumption patterns can be divided into three types: “animal-based food consumption” pattern (the proportion of animal food in the diet is more than 80%), which is the dominant pattern in most European and American countries; “plant-based food consumption” pattern (the proportion of plant food in the diet is more than 90%), which is the dominant pattern in most Asian countries; and “balanced animal and plant consumption” pattern (50% animal food and 50% plant food in the diet), which is the dominant pattern in most countries with well-balanced agricultural and livestock systems, such as Japan and Sweden. In 2016, the proportional consumption of animal-oriented food in China was only 15.19%, while in Bayannur, it was even lower (10.77%), and far below the consumption level in developed countries. Therefore, it is necessary to clarify the impact of changing food consumption patterns on N losses due to the possible increase in the consumption of “high N” foods in the future.

**Figure 4:**
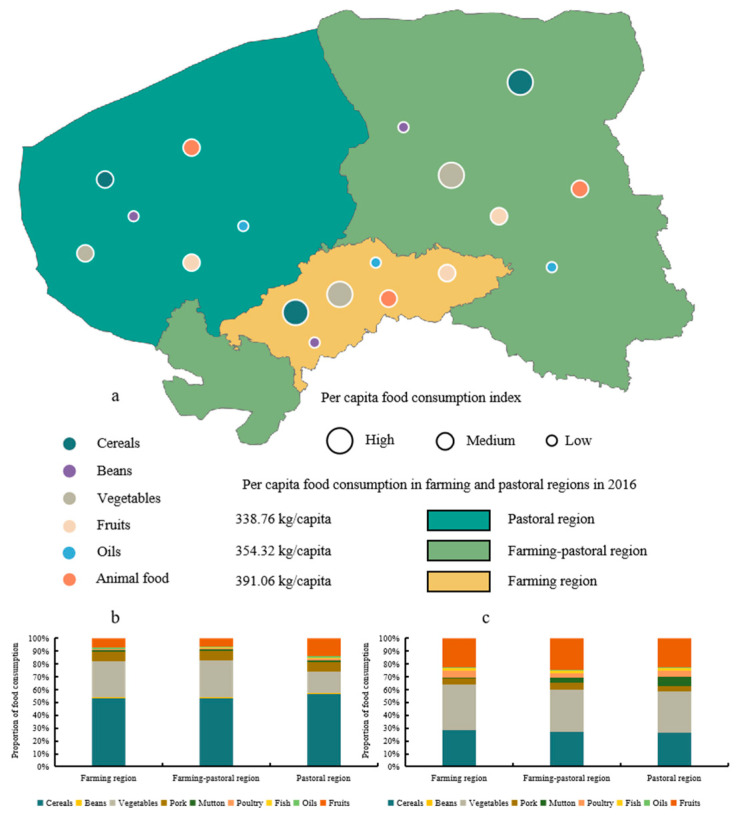
Per-capita food consumption and changes in the three regions of Bayannur from 2000 to 2016: (**a**) food consumption in Bayannur in 2016; (**b**,**c**) the proportion of food consumption in the three regions of Bayannur in 2000 and 2016, respectively.

Although the composition of food in the farming, farming–pastoral, and pastoral regions were similar in 2000, consumers were enthusiastic about high-carbohydrate foods. There were differences in the shifts in food consumption patterns in the three regions over the 16-year study period (Figure 4). The pastoral region had the most significant change in food consumption patterns. The proportional consumption of cereals decreased from 56.91% to 26.42%, while the proportional consumption of vegetables, fruits, and animal-oriented food consumed increased from 16.95%, 14.15%, and 10.10% to 31.88%, 22.69%, and 16.34%, respectively. In terms of animal-oriented food, the proportional consumption of mutton and poultry increased significantly from 15.20% and 15.98% to 44.42 and 29.35%, respectively. Compared with the pastoral region, there were more vegetable fields in the farming region, and the proportional consumption of vegetables was more than 25% between 2000 and 2016. Similarly to the pastoral region, the proportional consumption of cereals in the farming region decreased from 53.27% to 28.18%, while the proportional consumption of fruits increased from 7.15% to 22.63%. Likewise, consumers in the farming region were more likely to consume mutton and poultry than pork, and the proportional consumption of mutton and poultry increased from 17.28% to 57.22%. The change in food consumption patterns in the farming–pastoral region was intermediate between the other two regions, although food consumption patterns tended to shift from the “high carbohydrate and pork pattern” to the “high fiber and herbivore pattern”.

### 3.2. N Losses from the Food Supply in Bayannur

**Figure 5:**
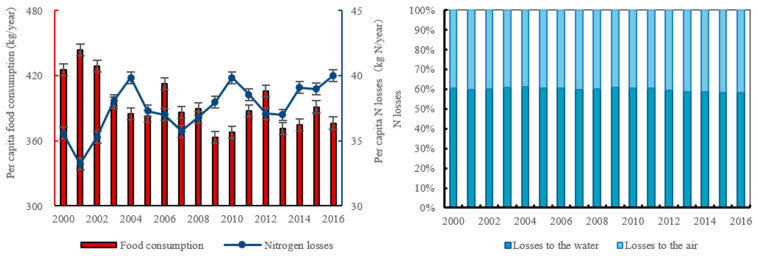
Variation of food consumption and N losses from 2000 to 2016.

Between 2000 and 2016, N losses to the environment from the food supply in Bayannur increased by 12.42% from 35.60 kg cap^−1^ (Figure 5). The average contribution of the plant-oriented and animal-oriented food supply to these losses was 53.39% and 46.61%, respectively. N losses to water accounted for most (58.25–61.11%) of the TN losses to the environment, while N losses to the air accounted for the smallest share (38.89–41.75%).

The N losses differed among the plant-oriented food items. During the study period, the N losses from the five crops increased by 13.65% from 19.69 kg N cap^−1^ (Table 2). Among the five crops, the N losses from the cereals gradually decreased by 47.74% from 8.57 kg N cap^−1^, while the beans decreased by 36.42% from 0.32 kg N cap^−1^. However, a significant increase was observed in the other three crops. Vegetables contributed N losses increased by 45.36% from 9.45 kg N cap^−1^, while fruits with similarly high N fertilizer requirements contributed N losses increased by 195.64% from 1.20 kg N cap^−1^. Notably, the N losses from the oil were small but increased rapidly, it increased by 172.05% from 0.16 kg N cap^−1^. Similarly, different animal-oriented food supplies were responsible for different N losses. The N losses to the environment from the four animals studied increased by 10.90% from 15.91 kg N cap^−1^. The change in food consumption patterns resulted in N losses from pork decreasing by 49.60% from 11.91 to 6.00 kg N cap^−1^. The other three animal-oriented foods all increased significantly. The mutton-contributed N losses increased by 132.01% from 3.06 kg N cap^−1^, while the poultry increased by 383.63% from 0.94 kg N cap^−1^. More dramatically, the N losses from the fish sharply increased by 511.97% from 0.17 kg N cap^−1^.

**Figure 6:**
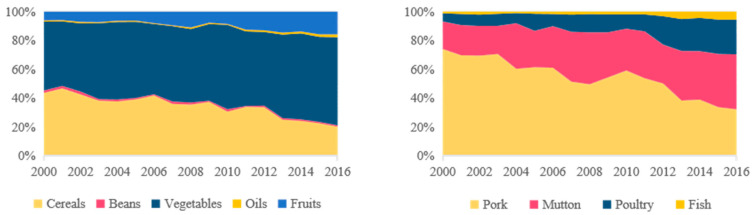
The proportion of N losses from different species among the plant- and animal-oriented food items from 2000 to 2016.

The contributions and changes were different for the five crops (Figure 6). Vegetables were responsible for the largest N losses among the plant-oriented food items, accounting for a maximum of 47.98% in 2000 and increasing to 61.37% in 2016. This was followed by cereals, which decreased from 43.54% to 20.02%. The change in fruits was also significant, with an increase from 6.08% to 15.81%. Beans and oils accounted for a smaller share of the TN losses, with their contributions remaining steady at 1%. Similarly, the contributions of the different animals to these losses were different in 2000 and 2016. The pork was responsible for the largest N losses among animal-oriented foods in 2000 (74.84%), while the contribution decreased to 34.01% in 2016. Over the study period, the proportion of N losses due to mutton and poultry production increased significantly from 19.27% and 5.89% to 40.31% and 25.58%, respectively. Interestingly, the increasing consumption of fish by consumers resulted in an increase in its share of N losses from 1.05% to 5.80%.

Although the contributions of different food items to N losses to air and water did not differ largely among the three regions, there were large differences in the amounts and changes of the N losses (Table 3). Notably, the N losses to the environment in the pastoral region increased sharply by 103.79% from 22.67 to 46.20 kg N cap^−1^ over the 16 years studied. The most significant change between the two food categories was for animal-oriented food, for which N losses increased by 142.57% from 11.98 kg N cap^−1^, while the losses from plant-oriented food grew more slowly (10.51%). It is necessary to emphasize that the N losses caused by mutton surprisingly increased by 444.90% from 3.43 kg N cap^−1^ because of the dramatic increase in the consumption of mutton by consumers. Compared to the pastoral region, the change in TN losses was smaller in the farming–pastoral (9.45%) and farming (8.44%) regions. In the farming region, N losses increased from 34.24 to 37.13 kg N cap^−1^, while plant-oriented food grew at a higher rate (11.07%) than animal-oriented food (5.00%). The situation was complex in the farming–pastoral region, although the N losses from plant-oriented food decreased by 7.22% from 20.08 kg N cap^−1^, the N losses from animal-oriented food increased excessively (30.52%), which increased the N losses from 15.89 to 20.74 kg N cap^−1^.

The FNC value can be interpreted as pollution of the environment to satisfy consumers’ food consumption demands. In the present study, the FNC value increased by 58.18% from 10.02 kg kg^−1^ in Bayannur City due to the increased consumption of vegetables, fruits, poultry, and mutton. The results above show that the diets in all three agricultural regions in Bayannur shifted over the study period to a food consumption pattern with higher N losses. The change in food consumption patterns resulted in a reshaping of regional demand, which indirectly affected the structure of agriculture. Vegetable, fruit, and animal-oriented food production are human activities resulting in large levels of N pollution. The high N fertilizer demand for fruits and vegetables, and the direct discharge of manure from sheep and poultry will lead to a continued increase in N losses in the future. Therefore, it is necessary to introduce measures to decrease N losses from the food supply under the new food consumption patterns.

### 3.3. Food and N Management

Food production and consumption were influenced by ethnic structure, consumer price index, resident education level, and so on. The change in dietary structure and the resulting food species choice have different degrees of N load to the environment [11]. As crop species, i.e., wheat and corn require relatively less N fertilizer. The increased demand for vegetables and fruits in the study area (Figure 3) resulted in enhanced environmental N loss but more micronutrient and vitamin intake. Therefore, nutritional security also needs to be concerned before diet management was proposed.

In 2016, the per-capita consumption of vegetables in Bayannur was 130 kg cap^−1^, higher than the recommended value (109.50 kg cap^−1^) of the Dietary Guidelines for Chinese Residents [48], which means that even a 15.77% reduction in vegetable consumption can meet the nutritional needs of consumers. Similarly, the per capita consumption of fruits was 14.97% higher than the recommended consumption of the guidelines. To ensure food security and take into account dietary nutrition, the dietary structure of residents in Bayannur is in urgent need of adjustment, and there is great room for improvement in reducing N loss during the 14th Five-Year Plan.

The measures developed to ensure food needs and environmental protection are: (1) increasing the wheat planting area by more than 50% from 35,457 ha year^−1^; (2) using inter-planting techniques to improve planting efficiency; (3) keeping the corn planting area at about 320,000 ha year^−1^, including at least 28% for both grain-feeding and silage. Because research shows that the increased demand for animal-oriented food caused a rise in N losses [11,12]. Increasing the proportion of grass feed is one important way to reduce N losses; (4) precision agriculture is recommended in the future. The traditional crop-planting method has a large demand for water usage and low N fertilizer use efficiency [12]. Optimizing the soil environment, improving water and N fertilizer management, and maximizing dense tolerant varieties can solve the problem of inadequate water and fertilizer utilization in the planting pattern, which can effectively improve production and reduce the N losses of crops. In addition, reducing herbicide application and improving the breeding technique may reduce N losses. The case study in Bayannur proved more than 45% of the herbicide was reduced in the corn field, and more than 60% in the sunflower field, which greatly reduced the pollution of the non-point sources [49]. Finally, using northern trade ports to import high-demand foods is also a way to reduce N losses.

## 4. Conclusions

This study integrated the CFSD and NUFER models to link food consumption transformation and N losses. The food consumption patterns, the contribution of different foods to the overall N losses, and FNC were estimated for different agricultural regions using a case study of Bayannur City in the YRB from 2000 to 2016. It was found that the shift in food consumption patterns in Bayannur over the study period increased N losses, which were more significant in pastoral regions. The implementation of effective control measures would reduce N losses. The main conclusions were as follows.

(1) From 2000 to 2016, Bayannur’s food consumption pattern changed from a “high carbohydrate and pork pattern” to a “high fiber and herbivore pattern”, which reflected the shift from low to high N consumption. The proportional consumption of cereals and pork decreased from 64.00% to 32.48%, and the proportional consumption of vegetables, fruits, livestock, poultry, and mutton increased from 34.45% to 62.95% during the study period. The expanded production of these foods led to increasing N losses in agricultural production.
(2) The food consumption decreased by 11.55% from 425.41 kg N cap^−1^, whereas the N losses increased by 12.42% from 35.60 kg N cap^−1^. The average share of the plant- and animal-oriented food supply in these losses was 53.39% and 46.61%, respectively. The N losses to the water bodies dominated 58.25–61.11% of the total losses to the environment. Over the study period, the FNC increased by 58.18% from 10.02 kg kg^−1^ due to the increased consumption of vegetables, fruits, poultry, and mutton.
(3) Obvious differences in the food consumption patterns and N losses in Bayannur’s farming, farming–pastoral, and pastoral regions were found. The changes in N losses were most significant in the pastoral region. The N losses to the environment in the pastoral region increased sharply by 112.33% from 22.75 to 48.31 kg N cap^−1^ over the past 16 years. The most significant change among the two food categories was found for animal-oriented food, which increased by 142.60% from 11.98 kg N cap^−1^. The N losses due to mutton consumption surprisingly increased by 737.76% from 3.43 kg N cap^−1^, which was due to the substantial increase in mutton consumption by consumers.

Grasslands provide approximately 70% of the fodder consumed by livestock worldwide, while the livestock reared in countries or regions mainly dominated by grasslands also serve as the primary source of food and income for residents. The moderate use of N and improved N use efficiencies need to be achieved. The outcomes of our study supported the formulation of effective nutrient management strategies in crop production to reduce N losses and support Agricultural Green Development in the Hetao Irrigation District. In future research, the model could be used to improve the N use efficiency in the grassland area, which could then serve as an example for other cities in China.

## 5. Limitations and Future Research

The low volume of food trade in Bayannur resulted in no quantification of the effect of import and export food consumption on N losses, which was different from the results of studies with frequent trade at the national scale or even at the provincial scale. Future studies can distinguish the effects of local and cross-regional consumption on N losses by taking the study area with more trade. Secondly, the differences in crop production in various soil environments have not been distinguished from the soil properties perspective when quantifying crop production. However, the physicochemical indicators of different soils greatly affect crop production, so future studies could distinguish based on agroecological zoning or use GIS to invert different crop production regions to investigate the differences in N losses. In addition, the food security mentioned above only considers quantity, without considering aspects of food quality, accessibility, affordability, and utilization. Moreover, vegetables and fruits are the food supply to ensure nutritional security, so nutritional security should be considered while protecting the environment. Due to the lack of statistical data during the study, the proportion of fodder grains and grasses supplied was not separated when quantifying animal-oriented food supply. In the discussion, it has been mentioned that the government focuses on the planting of a variety of fodder grains and grasses; therefore, the contribution of N losses from different types of fodder grains and grasses can be quantified in the future. Finally, crops are planted, and policies are implemented with changing needs. Further studies that include farmer questionnaires would result in more reasonable food and N management.

## Figures and Tables

**Table 1:** Results of the parameter sensitivity and uncertainty analysis.

Parameters	Unit	Maximum Values	Minimum Value	Average Value	Standard Deviation	Coefficient of Variation	|I|
Cereals N application	kg N·ha^−1^ year^−1^	252.00	228.00	240.14	6.90	2.90	0.29
Vegetables N application	kg N·ha^−1^ year^−1^	366.24	331.37	348.89	10.06	2.89	0.24
Weight of pig	kg	105.00	95.00	100.04	2.91	2.87	0.33

**Table 2:** The N loss from food species consumed by residents in Bayannur City from 2000 to 2016.

Categories Items Year	N Loss (kg N cap^−1^)										
	Plant-Oriented Food						Animal-Oriented Food				
	Cereals	Beans	Vegetables	Oils	Fruits	Total	Pork	Mutton	Poultry	Fish	Total
2000	8.57	0.32	9.45	0.16	1.20	19.69	11.91	3.06	0.94	0.17	15.91
2001	9.15	0.32	8.81	0.16	1.15	19.59	9.66	2.91	1.08	0.21	13.64
2002	8.40	0.38	9.40	0.18	1.41	19.76	11.00	3.26	1.26	0.31	15.53
2003	7.50	0.20	10.41	0.12	1.43	19.67	13.19	3.64	1.58	0.26	18.41
2004	7.37	0.25	10.58	0.14	1.24	19.59	12.34	6.47	1.46	0.19	20.26
2005	7.49	0.18	10.18	0.12	1.21	19.17	11.27	4.65	2.19	0.26	18.11
2006	7.99	0.15	9.38	0.10	1.53	19.14	11.08	5.24	1.55	0.28	17.86
2007	7.06	0.31	10.31	0.10	1.86	19.63	8.44	5.69	1.97	0.33	16.10
2008	6.74	0.26	9.74	0.19	2.09	19.03	8.95	6.51	2.31	0.30	17.76
2009	6.70	0.13	9.61	0.15	1.38	17.97	11.06	6.38	2.56	0.36	20.00
2010	6.31	0.33	12.15	0.11	1.77	20.67	11.54	5.67	1.96	0.35	19.18
2011	6.35	0.10	9.75	0.20	2.35	18.75	10.83	6.57	2.40	0.37	19.80
2012	6.64	0.20	10.15	0.21	2.56	19.76	8.95	4.83	3.55	0.55	17.33
2013	5.20	0.21	12.16	0.27	3.05	20.89	6.49	5.84	3.77	0.86	16.10
2014	5.22	0.21	12.98	0.27	2.98	21.67	7.07	6.13	4.22	0.78	17.42
2015	5.06	0.20	13.36	0.37	3.56	22.56	5.84	6.42	4.17	0.94	16.43
2016	4.48	0.20	13.73	0.43	3.54	22.38	6.00	7.11	4.53	1.02	17.64

**Table 3:** Comparison of the N loss from different food species in the three regions in 2000 and 2016.

Categories	Items	N loss (kg N cap^−1^)					
		2000			2016		
		Farming	Farming–Pastoral	Pastoral	Farming	Farming–Pastoral	Pastoral
plant-oriented food	cereals	9.80	9.33	4.69	4.89	4.18	3.85
	beans	0.03	0.02	0.02	0.02	0.01	0.02
	vegetables	8.96	10.20	5.17	15.00	12.80	11.81
	oils	0.07	0.04	0.03	0.04	0.04	0.02
	fruits	0.57	0.49	0.77	1.64	1.61	1.42
	total	19.43	20.08	10.69	21.58	18.63	17.14
animal-oriented food	pork	11.12	9.90	6.97	6.24	6.32	4.96
	mutton	2.53	5.20	3.43	2.07	10.10	18.69
	poultry	1.16	0.79	1.58	7.24	4.32	5.41
	fish	0.17	0.16	0.25	1.06	1.03	0.86
	total	14.81	15.89	11.98	15.55	20.74	29.06

## Data Availability

The data used to support the findings of this study can be made available by the corresponding author upon request.
